# Hidradenocarcinoma of the Left Knee in a 55-Year-Old Woman: A Case Report

**DOI:** 10.7759/cureus.27395

**Published:** 2022-07-28

**Authors:** Muhammad Abu Zar Ghaffari, Faraz Saleem, Faiqa Zahoor, Saima Batool, Usman Ismail, Muhammad Usman, Hafiz Fahad Ullah Saeed

**Affiliations:** 1 Department of Internal Medicine, Akhtar Saeed Medical and Dental College, Lahore, PAK; 2 Department of Internal Medicine, Al Ameen Medical College, Vijayapura, IND; 3 Department of Internal Medicine, Bolan Medical College, Quetta, PAK; 4 Department of Internal Medicine, Mayo Hospital, Lahore, PAK

**Keywords:** adnexal tumor, hidradenoma, clear cell, soft tissue, malignant, hidradenocarcinoma

## Abstract

The occurrence and scientific reporting of benign adnexal tumors arising from the eccrine and apocrine sweat glands, hair follicles, and pilosebaceous components of the skin is very rare. Even though they are uncommon, these long-standing benign lesions can transform into their malignant counterparts, which can be exceedingly difficult to treat because malignant lesions are linked to higher rates of morbidity and mortality. Here, we present a rare instance of primary hidradenocarcinoma of the left knee in a 55-year-old lady.

## Introduction

Hidradenocarcinoma is a rare malignant adnexal tumor that arises from eccrine sweat glands, accounting for 6% of all malignant eccrine tumors [1]. The most common site of origin is the head and neck region, with rarer cases presenting in the extremities [2]. The most common age of presentation is the fifth to the seventh decade of life. Females are more commonly affected [3]. There are currently no known risk factors. It usually presents as a slow-growing, nodular, solid, and cystic cutaneous mass associated with serous discharge or ulceration [4]. The benign histopathological variants are clear cell, solid cystic, mucinous, poroid, and pigmented nodular hidradenoma [4,5]. The benign lesions can undergo malignant transformation, though very rarely [6].

Hidradenocarcinoma has an aggressive course, with high rates of recurrence and metastasis. It carries a poor prognosis due to metastasis to lymph nodes, bones, or visceral organs, and often causes death, with a reported five-year survival rate of approximately 30%. Histopathology remains the gold standard for its diagnosis [7,8]. We describe a case of hidradenocarcinoma presenting as a primary neoplasm on the left popliteal fossa.

## Case presentation

**Figure 1:**
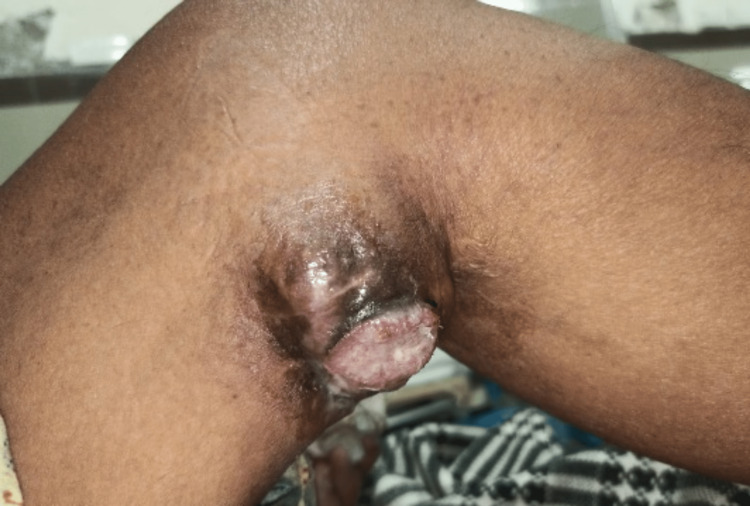
Photograph showing a soft tissue growth, centrally ulcerated with pigmentation.

Our patient is a 55-year-old female who came to the hospital’s surgical outpatient department (OPD) complaining of a swelling on the medial aspect of her left knee. A year prior, the lesion was discovered as a small lump that had grown larger over time and was now accompanied by pain, ulceration, and bloody discharge. Physical examination revealed a soft tissue mass measuring 7 × 9 cm on the medial aspect of the left knee. The mass was soft in consistency, mobile in all planes, and tender. In addition to the primary ulcers, the overlying skin was noted to be hyperpigmented. Figure 1 displays these findings. The sensorimotor status of the surrounding skin was intact, and no regional lymph nodes were palpable.

**Figure 2:**
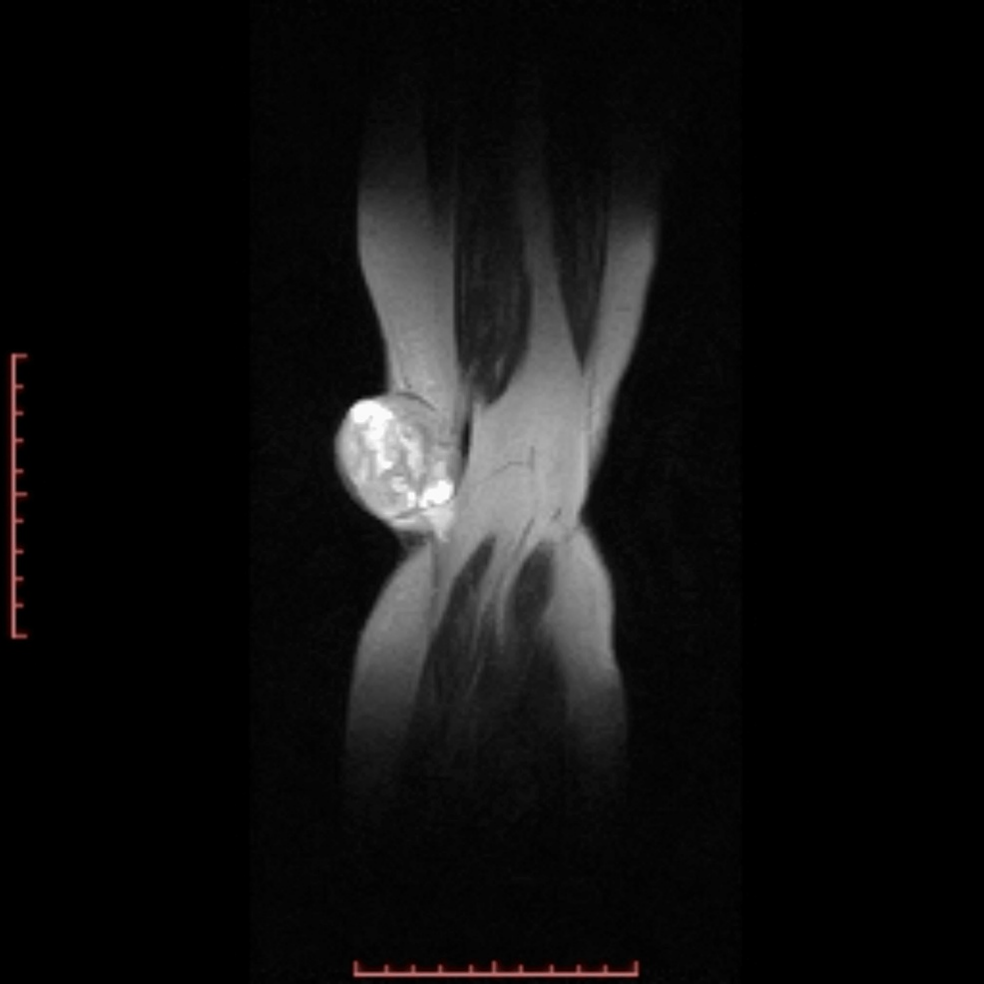
T2 coronal slice showing a well-circumscribed mass predominantly solid with cystic component compared with T1 slice, with no local invasion.

**Figure 3:**
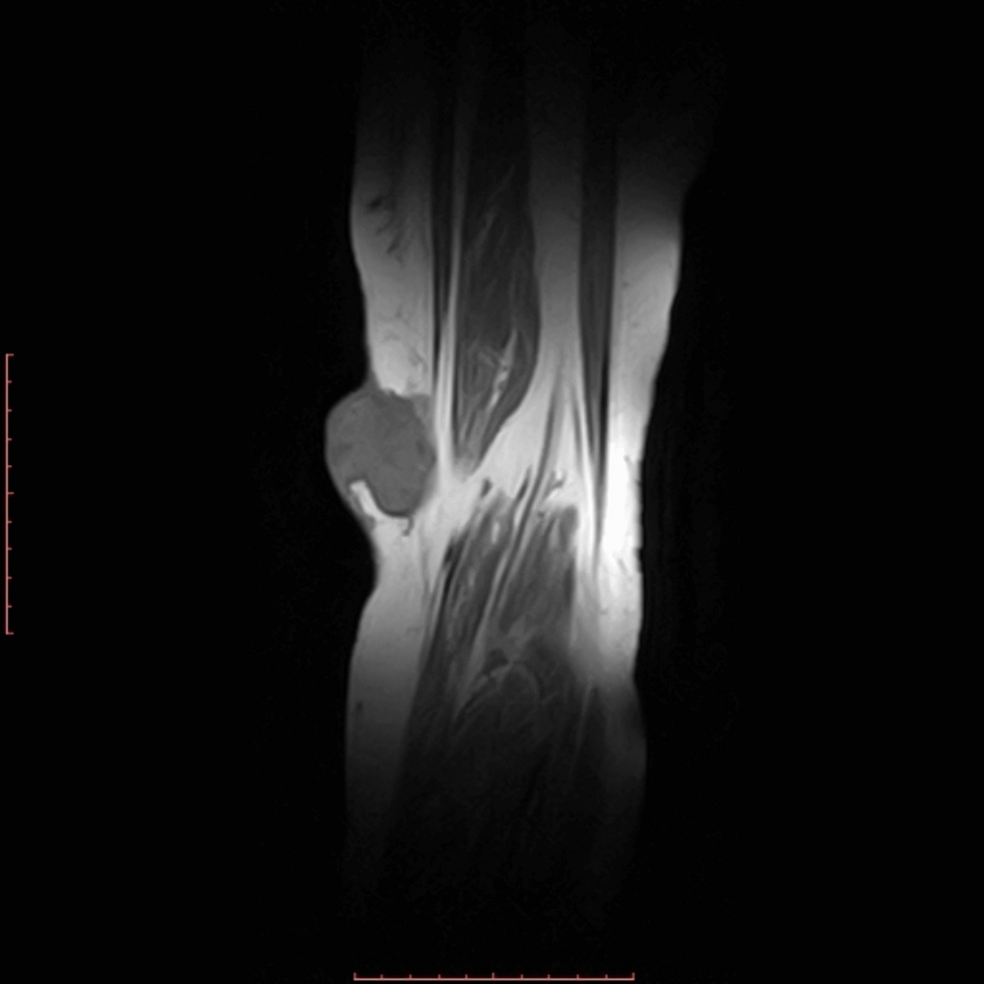
Magnetic resonance imaging: T1 coronal slice showing a well-circumscribed mass, both solid and cystic components of the lesion in the subcutaneous plane of the medial popliteal fossa can be seen. No invasion of soft tissue, joint space, or bone.

Plain radiograph demonstrated no involvement of the underlying structures. Magnetic resonance imaging (MRI) of the left popliteal fossa in the subcutaneous plane showed a well-defined signal intensity mass measuring 4.4 × 4.6 cm consisting of both solid and cystic components. The solid component was noted to produce hypointense signals on both T1 and T2-weighted images, while the cystic component produced hypointense signals on T1 and hyperintense signals on T2-weighted images. Medially, the mass was abutting the tendons of semitendinosus and gracilis muscles. However, as depicted in Figure 2 and Figure 3, there was no evidence of an invasion of the skeletal or muscle tissue.

**Figure 4:**
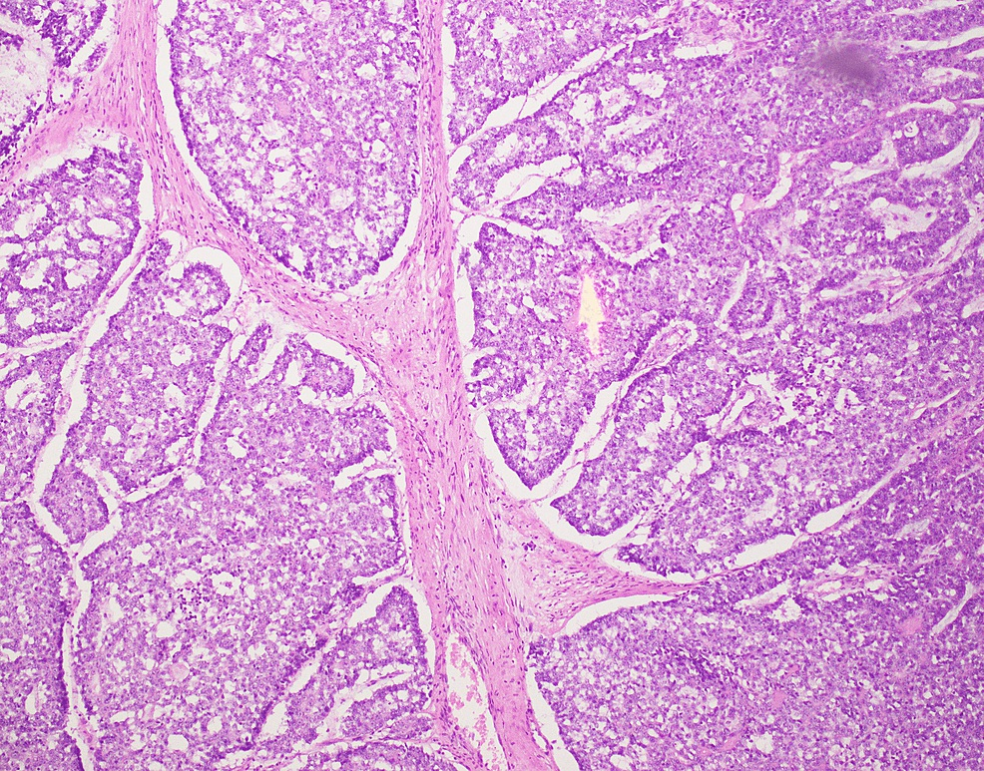
Histopathology of the resected specimen shows a circumscribed multilobulated neoplasm of polygonal cells with basophilic cytoplasm and clear cells. Tumor cells exhibit moderate atypia. Scattered mitoses are also seen.

A full body computed tomography (CT) scan was ordered, which showed no metastases of the primary lesion. A differential diagnosis of schwannoma, benign skin tumors, and tumors of mesenchymal origin was made. The patient underwent wide local excision of the mass, and the histopathological examination indicated a well-circumscribed, multilobulated neoplasm of polygonal cells with basophilic cytoplasm and clear cells. Tumor cells displayed moderate atypia. Scattered mitoses were also seen (Figure 4).

The surgical margin was free of tumor cells. At the six-month follow-up, no recurrence or any evidence of distant metastases was observed.

## Discussion

Hidradenocarcinoma is the malignant counterpart of hidradenoma, an eccrine sweat gland tumor. This uncommon phenomenon is referred to by a variety of medical terms, including clear cell hidradenocarcinoma, malignant clear cell myoepithelioma, clear cell eccrine carcinoma, primary mucoepidermoid cutaneous carcinoma, and malignant acrospiroma [3,4]. It has been estimated that 6-7% of benign neoplasms will mysteriously convert into malignant tumors [9]. Contrary to an Indian study that reported men between the ages of 50 and 70 were somewhat more likely to develop hidradenocarcinoma, our female patient, aged 55, developed the disease as a primary instance [8].

The presenting complaint of our patient was an ulcerated swelling on the medial aspect of the left popliteal fossa. Extremities are the least likely locations for this malignancy to arise; the head and anterior trunk are where it occurs more frequently [6].

The ulcerated, nodular, dermal swelling with or without adhesion to the overlying skin is a consistent finding that has been recorded in a study similar to ours [3]. The majority of individuals with this type of swelling have an asymptomatic clinical course throughout their disease history, with pain and bleeding upon contact as the most prevalent symptoms. These tumors often have a diameter of 1-5 cm [8]. However, due to the patient’s delayed presentation to the clinical setting, a bigger mass measuring 7-9 cm with overt ulceration was observed. In our case study, the investigation revealed a well-defined mass that was partially solid and partially cystic without any evidence of invasion. A Japanese case report, however, described this tumor as a poorly defined lesion with homogeneity and diffuse enhancement [6]. Another study revealed a low signal intensity of the same lesion on T1 and T2-weighted imaging [7].

Squamous cell cancer, basal cell carcinoma, metastatic clear cell carcinoma of the lungs and kidneys, dermatofibrosarcoma, eccrine porocarcinoma, and eccrine ductal carcinoma were the differential diagnoses under investigation [1,7,8]. The histological evaluation of such swellings is crucial to determine the prognosis. The patient in our case study had hidradenocarcinoma, which was evident by the polygonal cells’ basophilic cytoplasm, moderate atypia, and scattered mitoses. Similar findings were reported in a Portuguese investigation, which also found high nuclear pleomorphism, necrotic regions, high mitotic rates, and perineural and lymphovascular invasion [2].

However, another study also revealed that hidradenocarcinoma frequently contains epidermoid cells, signet ring cells, and sarcomatoid alterations [8]. After thorough lab analysis, our patient’s immunohistochemistry included positive results for the carcinoembryonic antigen, cytokeratin 7, and epithelial membrane antigen, similar to that of an Indian case study. Additionally, a 40% positivity rate for Ki67 was also recorded [8].

The patient received broad local excision with a 2 cm safe marginal surgical dissection. Regional lymph nodes were left intact. In Japan, a similar surgical approach was used to treat this malignancy in the lower leg. Because prophylactic lymph node dissection has not been explicitly recommended in any such study to date, it was not performed in our patient either [6]. High mitotic index, vascular invasion, and tumor depth greater than 7 mm have been identified by Robson et al. as the factors associated with a poor prognosis [10]. The local recurrence rate for hidradenocarcinoma is significant at 50% [7]. Our patient was monitored twice monthly for six months, and throughout that period, she showed good mobility, with no signs of local recurrence or abnormal postoperative regional neurovascular functions.

## Conclusions

Hidradenocarcinoma is a well-circumscribed malignant transformation of hidradenoma, a benign eccrine sweat gland tumor. It is treated surgically with wide excision. It is recommended to be followed up properly with a high suspicion of recurrence and metastatic disease.
